# Vibralactone Biogenesis-Associated Analogues from Submerged Cultures of the Fungus *Boreostereum vibrans*

**DOI:** 10.1007/s13659-017-0147-5

**Published:** 2017-12-05

**Authors:** He-Ping Chen, Meng-Yuan Jiang, Zhen-Zhu Zhao, Tao Feng, Zheng-Hui Li, Ji-Kai Liu

**Affiliations:** 1 School of Pharmaceutical Sciences, South-Central University for Nationalities, Wuhan, 430074 People’s Republic of China; 2 Key Laboratory of Chemistry in Ethnic Medicinal Resources, State Ethnic Affairs Commission & Ministry of Education, School of Ethnic Medicine, Yunnan Minzu University, Kunming, 650504 People’s Republic of China

**Keywords:** Basidiomycete, *Boreostereum vibrans*, Vibralactone, Structure revision, Snatzke’s method

## Abstract

**Abstract:**

A scale-up fermentation of the fungus *Boreostereum vibrans* facilitated the isolation of six new vibralactone biogenesis-associated analogues, namely vibralactamide A (**1**), vibralactone T (**2**), 13-*O*-lactyl vibralactone (**3**), 10-*O*-acetyl vibralactone G (**4**), (11*R*,12*R*)- and (11*S*,12*R*)-vibradiol (**5**, **6**). Their structures were established via extensive spectroscopic analyses, specific optical rotation comparison, and Snatzke’s method. The biosynthetic pathway for vibralactamide A was postulated. The absolute configuration of vibralactone B was revised by single crystal X-ray diffraction analysis. This work puts the divergent vibralactone biosynthesis pathway one step further and expands the structural diversity of vibralactone-associated compounds.

**Graphical Abstract:**

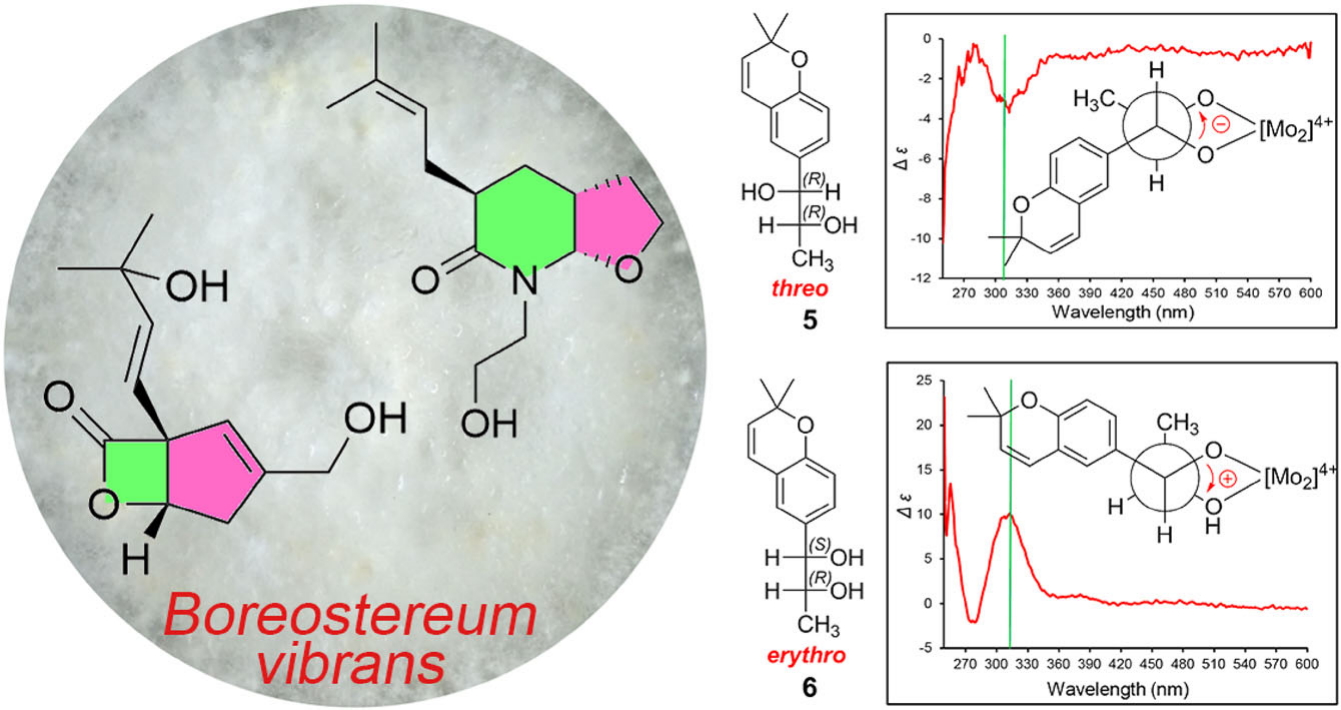

**Electronic supplementary material:**

The online version of this article (10.1007/s13659-017-0147-5) contains supplementary material, which is available to authorized users.

## Introduction

Natural products from higher fungi origin are irreplaceable sources of architecturally distinct scaffolds for drug discovery [1, 2]. The fungus *Boreostereum vibrans* is featured by producing a lipase inhibitor named vibralactone which harboring an unusual *β*-lactone group. In recent years, endeavors to explore the potential of producing diverse metabolites of *B. vibrans* by scale-up fermentation and/or changing culture medium compositions led to the isolation of plenty of bioactive vibralactone congeners and vibralactone biosynthetic-associated compounds [3–11], including vibralactoximes A–P which represented the first class of natural oxime esters [3]. The biosynthesis of vibralactone and divergent biosynthetic pathway for skeletally distinct vibralactone-associated compounds were illuminated [12, 13]. It was proved that all these compounds shared the biosynthetic precursor 3-prenyl-4-hydroxybenzylalcohol. An FAD-binding monooxygenase (VibMO1) from *B. vibrans* that converted prenyl 4-hydroxybenzoate into prenylhydroquinone was also characterized for the first time.

**Fig. 1:**
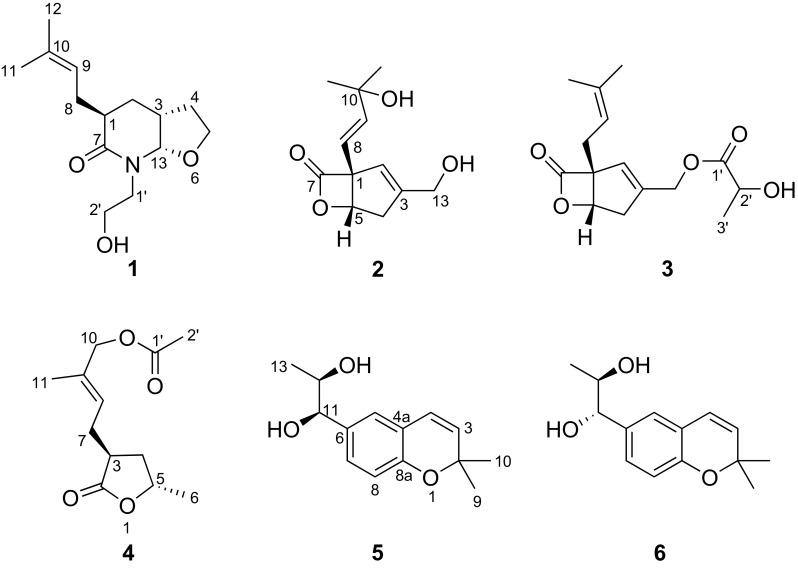
Chemical structures of compounds **1**–**6**

This study, as part of our ongoing interest in bioactive metabolites from higher fungi, focused on the trace compounds of scale-up submerged cultures of *B. vibrans*. As a result, six previously undescribed vibralactone biogenesis-associated analogues were identified. Herein, we report the isolation, structure elucidation, and absolute configuration determination via comparison of specific optical rotations and application of Snatzke’s method of these new compounds (Fig. 1). We also make a revision of the absolute configuration of vibralactone B.

## Results and Discussion

**Fig. 2:**
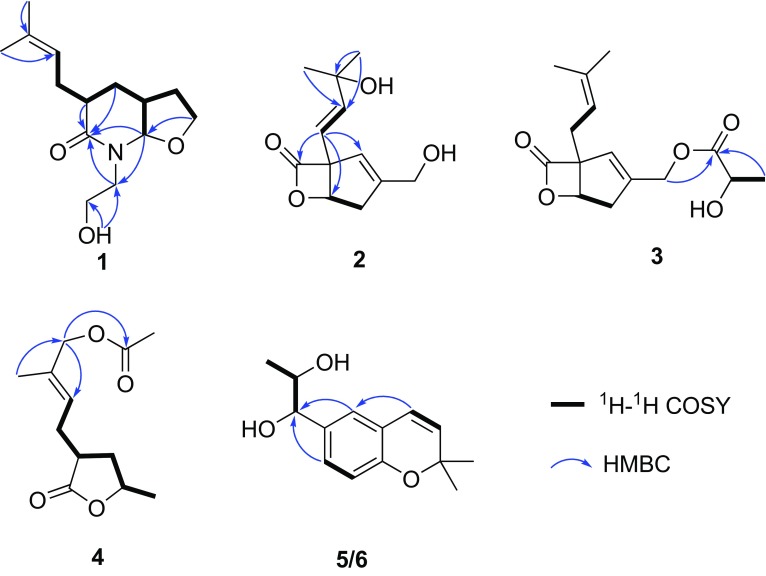
Selected ^1^H−^1^H COSY and HMBC correlations of compounds **1**–**6**

Compound **1** was obtained as colorless oil. It had a molecular formula C_14_H_23_O_3_N as deduced from the HREIMS molecular ion peak at *m/z* 253.1672 [M]^+^ (calcd for 253.1678, C_14_H_23_O_3_N), indicating four degrees of hydrogen deficiency. The IR spectrum absorption bands at 3447 and 1637 cm^−1^ indicated the presence of hydroxy and carbonyl groups, respectively. The 1D NMR spectroscopic data (Tables 1 and 2) displayed fourteen carbon signals ascribable to two methyl singlets, six methylenes (two oxygenated), four methines (one olefinic, one oxygenated), and two quaternary carbons. The presence of a carbonyl (*δ*_C_ 173.8, C-7) and a double bond (*δ*_C_ 123.2, C-9; 133.5, C-10) accounting for two degrees of unsaturation suggesting two cycles in **1**. Further elucidating the 2D NMR spectra allowed the completion of planar structure of **1**. The HMBC correlations (Fig. 2) from the two methyl singlets (*δ*_H_ 1.60, 1.68) to C-9 and C-10, along with the ^1^H–^1^H COSY correlations between H-8 (*δ*_H_ 2.50, 2.13) and H-9 (*δ*_H_ 5.14) suggested the existence of an isopentenyl group. Analysis of the ^1^H–^1^H COSY spectrum allowed the following sequential connectivity from C-8 to C-5, from C-3 to C-13 (Fig. 2). The HMBC correlations from H-8 to C-1 (*δ*_C_ 38.3), C-2 (*δ*_C_ 28.9), and C-7 revealed that the carbonyl group connected to C-1. The key HMBC correlations from H-13 (*δ*_H_ 5.17) to the typical oxygenated methylene C-5 (*δ*_C_ 65.7) and carbonyl C-7 as well as the chemical shift of C-13 (*δ*_C_ 91.6) [14] led to the assignments of an ether bond between C-5/C-13, and a nitrogen atom between C-7/C-13, which constructed a hexahydrofuro[2,3-*b*]pyridin-6(2*H*)-one scaffold. The remaining two carbons (C-1′, *δ*_C_ 49.7; C-2′, 61.5) was assigned to a 2′-hydroxyethyl substituted at the nitrogen atom as supported by the ^1^H–^1^H COSY correlations between H-1′ (*δ*_H_ 3.61, 3.38) and H-2′ (*δ*_H_ 3.61), and HMBC correlations from H-1′ to C-7 and C-13. When changing NMR solvents from acetone-*d*_6_ to DMSO-*d*_6_, the resultant 2′-OH proton signal at *δ*_H_ 4.66 (t, *J* = 5.6 Hz) and less overlapped signals reinforced the above assignments.

**Table 1:** ^1^H NMR spectroscopic data for compounds **1**−**6** (600 MHz)

No.	**1** ^a^	**1** ^b^	**2** ^a^	**3** ^c^	**4** ^a^	**5** ^c^	**6** ^a^
1	2.34, m	2.30, m					
2	1.65, ddd (13.6, 11.0, 5.4) 1.81, ddd (13.6, 4.6, 4.3)	1.56, ddd (13.6, 10.7, 5.3) 1.70, overlapped	5.64, s	5.68, s			
3	2.71, m	2.61, m			2.79, ddd (16.8, 7.7, 5.0)	5.68, d (9.8)	5.70, d (9.8)
4	2.14, m 1.82, m	2.08, m 1.73, m	2.66, d (18.7) 2.87, dd (18.7, 5.7)	2.72, d (19.0) 2.83, dd (19.0, 6.0)	2.12, ddd (12.8, 7.7, 7.7) 2.04, overlapped	6.36, d (9.8)	6.38, d (9.8)
5	3.84, dd (13.8, 13.8, 5.8) 3.69, br. dd (13.8, 7.8)	3.74, ddd (8.2, 8.1, 6.1) 3.64, ddd (8.2, 8.1, 6.5)	4.99, d (5.7)	4.89, d (6.0)	4.65, m	6.99, d (2.1)	7.04, d (2.0)
6					1.32, d (6.4), 3H		
7					2.46, m 2.31, m	7.07, dd (8.3, 2.1)	7.10, dd (8.2, 2.0)
8	2.50, br. ddd (14.3, 5.3, 5.3) 2.13, overlapped	2.41, m 2.05, overlapped	5.88, d (16.0)	2.43, dd (15.0, 7.5) 2.61, dd (15.0, 7.5)	5.50, t (7.5)	6.68, d (8.3)	6.66, d (8.2)
9	5.14, br. t (7.3)	5.10, t (7.3)	5.99, d (16.0)	5.17, t (7.5)		1.39, s, 3H	1.38, s, 3H
10					4.43, s, 2H	1.39, s, 3H	1.38, s, 3H
11	1.60, s, 3H	1.59, s, 3H	1.26, s, 3H	1.66, s, 3H	1.68, s, 3H	4.24, d (7.2)	4.44, d (4.0)
12	1.68, s, 3H	1.68, s, 3H	1.26, s, 3H	1.73, s, 3H		3.77, m	3.82, m
13	5.17, d (7.0)	5.15, d (7.0)	4.17, dd (15.6, 6.8) 4.20, dd (15.6, 6.8)	4.74, d (13.0) 4.79, d (13.0)		0.96, d (6.4), 3H	1.04, d (6.3), 3H
1′	3.61, overlapped 3.38, m	3.31, dd (13.8, 6.6, 6.6) 3.41, overlapped					
2′	3.61, overlapped, 2H	3.46, overlapped, 2H		4.31, q (6.9)	2.01, s, 3H		
3′				1.39, d (6.9), 3H			
2′-OH	3.84, overlapped	4.66, t (5.6)					
11-OH							4.12, br. s
12-OH							3.54, br. s
13-OH			4.22, t (6.8)				

^a^Recorded in acetone-*d*_6_

^b^Recorded in DMSO-*d*_6_

^c^Recorded in CD_3_OD

**Table 2:** ^13^C NMR Spectroscopic Data for Compounds **1**−**6** (150 MHz)

No.	**1** ^a^	**1** ^b^	**2** ^a^	**3** ^c^	**4** ^a^	**5** ^c^	**6** ^a^
1	38.3, CH	36.9	70.2, C	76.8, C			
2	28.9, CH_2_	27.7	121.2, CH	126.4, CH	178.7, C	77.3, C	76.6, C
3	35.0, CH	33.7	149.5, C	143.3, C	39.7, CH	132.1, CH	131.5, CH
4	30.8, CH_2_	29.6	37.8, CH_2_	38.7, CH_2_	34.8, CH_2_	123.4, CH	123.2, CH
4a						122.4, C	122.4, C
5	65.7, CH_2_	64.7	81.2, CH	80.0, CH	75.4, CH	126.2, CH	125.7, CH
6					21.3, CH_3_	135.6, C	135.9, C
7	173.8, C	172.2	172.5, C	174.6, C	29.0, CH_2_	128.9, CH	128.3, CH
8	29.8, CH_2_	28.9	119.4, CH	28.5, CH_2_	125.6, CH	116.9, CH	116.1, CH
8a						153.8, C	152.8, C
9	123.2, CH	122.2	143.2, CH	118.8, CH	133.9, C	28.1, CH_3_	28.1, CH_3_
10	133.5, C	132.5	76.4, C	137.2, C	69.8, CH_2_	28.2, CH_3_	28.1, CH_3_
11	17.9, CH_3_	17.8	30.2, CH_3_	18.2, CH_3_	14.1, CH_3_	79.9, CH	77.9, CH
12	26.0, CH_3_	25.7	30.2, CH_3_	26.1, CH_3_		72.8, CH	71.9, CH
13	91.6, CH	90.0	61.1, CH_2_	63.5, CH_2_		19.2, CH_3_	18.2, CH_3_
1′	49.7, CH_2_	47.3		176.0, C	170.8, C		
2′	61.5, CH_2_	58.7		68.0, CH	20.8, CH_3_		
3′				20.8, CH_3_			

^a^Recorded in acetone-*d*_6_

^b^Recorded in DMSO-*d*_6_

^c^Recorded in CD_3_OD

**Fig. 3:**
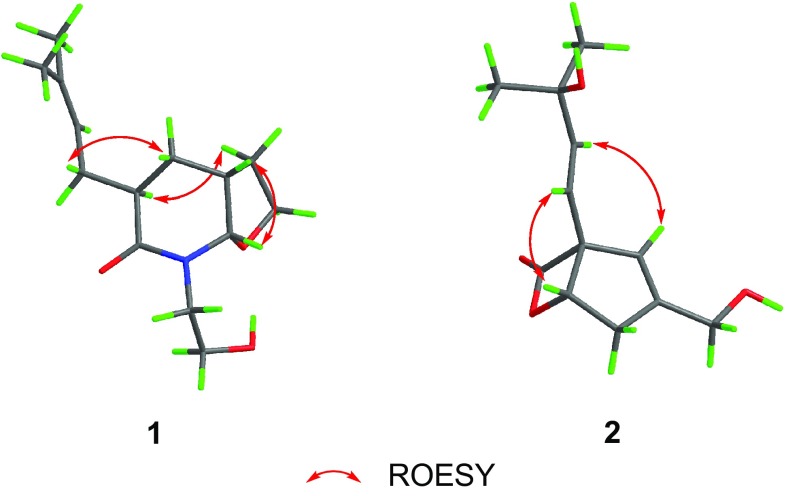
Selected ROESY correlations of compounds **1** and **2**

The relative configuration of compound **1** was established by interpretation of the ROESY spectrum. Evident ROESY signals between H-3 (*δ*_H_ 2.71)/H-13, H-1 (*δ*_H_ 2.34)/H-4*α* (*δ*_H_ 1.82), H-8a (*δ*_H_ 2.13)/H-2*β* (*δ*_H_ 1.81) indicated that the two rings were *cis*-fused and the isopentenyl group was *β*-oriented (Fig. 3). Therefore, compound **1** was established as shown in Fig. 1 and was trivially named as vibralactamide A.

**Scheme 1:**
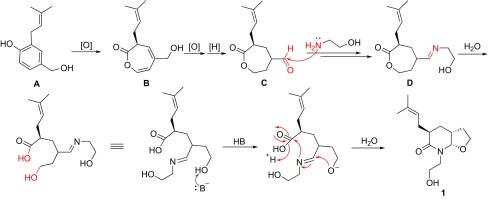
Proposed biosynthetic pathway for compound **1**

The biosynthetic pathway of vibralactamide A (**1**) was proposed as shown in Scheme 1. It was supposed that 3-prenyl-4-hydroxybenzylalcohol (**A**) underwent a C–C cleavage reaction to give 1,5-*seco*-vibralactone (**B**). The intermediate **B** was then subjected to cascade reduction and oxidation reactions to give **C**. The key intermediate **D** which formed by nucleophilic addition of **C** with 2-aminoethanol was subjected to hydroxylation/nucleophilic addition/dihydroxylation processes to afford compound **1**.

Compound **2** was obtained as colorless oil. The molecular formula was established as C_12_H_16_O_4_ by the sodium adduct ion peak at *m/z* 247.0937 [M + Na]^+^ (calcd for 247.0941, C_12_H_16_O_4_Na) in HRESIMS analysis. The 1D NMR spectra of compound **2** displayed signals of two overlapped methyl singlets, two methylenes, four methines (three olefinic, one was oxygenated), and four quaternary carbons (one carbonyl, one olefinic) (Tables 1 and 2). All these data exhibit similarities to those of vibralactone [8]. However, the presence of two *trans*-double bond protons (*δ*_H_ 5.88, *J* = 16.0 Hz, H-8; *δ*_H_ 5.99, *J* = 16.0 Hz, H-9), and the HMBC correlations from H-9 to Me-11/Me-12 (*δ*_C_ 30.1), C-10 (*δ*_C_ 76.4), and from H-8 to C-1 (*δ*_C_ 70.2), C-7 (*δ*_C_ 172.5), and C-2 (*δ*_C_ 121.2) suggested that the C9-C10 double bond in vibralactone shifted to the position C8–C9, while C-10 was substituted by a hydroxy in **2** (Fig. 2). This assignment was consistent with the HRESIMS result. The relative configuration of compound **2** was determined as 1R*,5S* via ROESY correlations between H-5 (*δ*_H_ 4.99) and H-8 (*δ*_H_ 5.88) (Fig. 3), and the absolute configuration was assigned as 1*R*,5*S* by comparison of the specific optical rotation value with those of vibralactone ($\left[ \alpha \right]_{\text{D}}^{25}$ −61.3 for **2**, $\left[ \alpha \right]_{\text{D}}^{26}$ −135.1 for vibralactone). Thus, compound **2** was elucidated as shown in Fig. 1, and was given the trivial name vibralactone T.

The ^13^C NMR and DEPT spectra (Tables 1 and 2) of the colorless oil compound **3** exhibited signals ascribable to three methyls, three methylenes, four methines, and five quaternary carbons (two carbonyls). These data show high resemblance to those of vibralactone [8]. Compared to vibralactone, the additional three carbons at *δ*_C_ 176.0 (C-1′), 68.0 (C-2′), 20.8 (C-3′) and corresponding protons at *δ*_H_ 1.39 (d, *J* = 6.9 Hz, H-3′), 4.31 (q, *J* = 6.9 Hz, H-2′) as well as the HMBC correlations from H-3′ to C-2′ and C-3′ indicated the presence of a lactyl moiety in **3**. The lactyl moiety attached at the oxygen atom at C-13 by the key HMBC correlation from H-13 (*δ*_H_ 4.74, 4.79) to C-1′. This assignment agreed with the HRESIMS result which gave a sodium adduct ion peak at *m/z* 303.1206 [M + Na]^+^ (calcd for 303.1203, C_15_H_20_O_5_Na). The absolute configuration of the vibralactone part was determined as 1*R*,5*S* via biogenetic consideration and evident ROESY correlations between H-5/H-8. However, attempt to settle the absolute configuration of chiral center C-2′ of the lactyl moiety by Mosher’s method failed. With (*R*)-MTPA derivative of **3** in hand, while the counterpart (*S*)-MTPA derivative was missing because of the insufficient quantity of compound **3**. Therefore, compound **3** was identified as 13-*O*-lactyl-vibralactone (Fig. 1).

Compound **4** was isolated as colorless oil. The 1D NMR data (Tables 1 and 2) displayed high similarities to those of vibralactone G [9], a γ-lactone derivative obtained from the same fungus but from different batches of cultures. Analysis of the 2D NMR spectra suggested that the 10-OH of vibralactone G was acetylated to give compound **4**, which supported by the HMBC correlation from H-10 (*δ*_H_ 4.43) and H-2′ (*δ*_H_ 2.01) to the carbonyl C-1′ (*δ*_C_ 170.8). The relative configuration of compound **4** was proved to be same with that of vibralactone G according to the ROESY correlations between H-3 (*δ*_H_ 2.79) and Me-6 (*δ*_H_ 1.32). Thus, compound **4** was identified as 10-*O*-acetyl vibralactone G (Fig. 1).

**Fig. 4:**
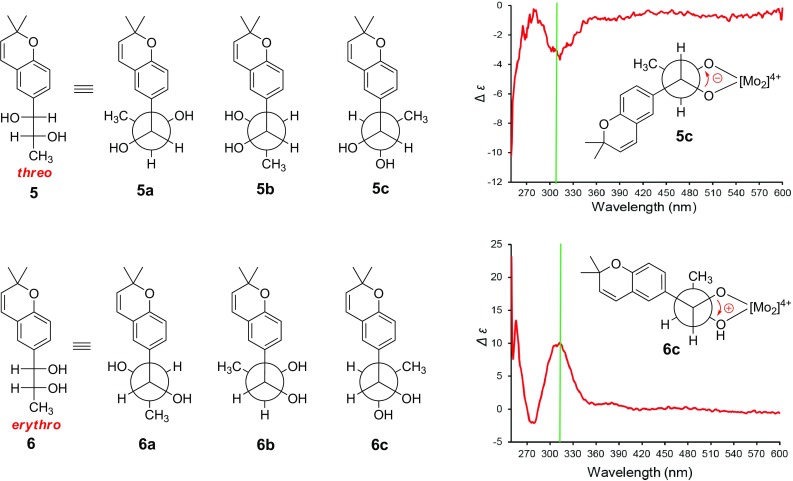
Fischer projections of compounds **5** and **6**; Newman projections of the possible conformers **5a**, **5b**, **5c** for **5**, and **6a**, **6b**, **6c** for **6**; and CD spectra of in situ formed Mo-complexes of **5** and **6** in DMSO after 0.5 h from dissolving in the 1:1.2 ligand-to-metal ratio (green lines marked the position of 310 nm)

Compounds **5** and **6** were purified as white powder. HREIMS analyses suggested that these two compounds had the same molecular formula of C_14_H_18_O_3_. The close NMR data (Tables 1 and 2) of these two compounds which showed resemblance to those of vibranether revealed that compounds **5** and **6** possessed same planar structure and were analogues of vibranether [10]. The absence of the methoxy signal in ^1^H NMR spectra of compounds **5** and **6** compared to that of vibranether enabled the establishment of planar structure of these two compounds as chromene vicinal diol derivatives (Fig. 1). The remarkable differences of chemical shifts at the position C-11, C-12, and C-13 though recorded in different NMR solvents suggested that these two compounds were C-11/C-12 diastereomers. Compounds **5** and **6** were determined as *threo* and *erythro* configurations by the coupling constants between H-11 and H-12 (*J* = 4.0 Hz for **5**; *J* = 7.2 Hz for **6**) as well as the chemical shifts of 13-methyls [15, 16]. The absolute configuration of the 11,12-diol moieties of compounds **5** and **6** were assigned using the in situ dimolybdenum CD method developed by Snatzke and Frelek [17, 18]. When adding dimolybdenum tetraacetate [Mo_2_(OAc)_4_] to solution of **5** and **6** in DMSO, respectively, metal complexes were generated as an auxiliary chromophore. The Cottonogenic diol derivatives are constrained into a chiral, *gauche* arrangement with the bulkier substituents adopt a pseudo-equatorial position pointing away from the remaining portion of the complex. The signs of band II (310 nm) are same with the O–C–C–O dihedral angle, and are most safely related to the absolute configuration of the diol. As shown in Fig. 4, the positive and negative Cotton effects at 310 nm of compounds **5** and **6** metal complexes corresponding to the clockwise and counter-clockwise of the O–C–C–O dihedral angles, respectively, which led to the unambiguously assign of the absolute configurations of **5** and **6** as 11*R*,12*R* and 11*S*,12*R*, respectively [19, 20]. Therefore, compounds **5** and **6** were elucidated as shown in Fig. 1 and were given the respective names (11*R,*12*R*)- and (11*S,*12*R*)-vibradiol.

**Fig. 5:**
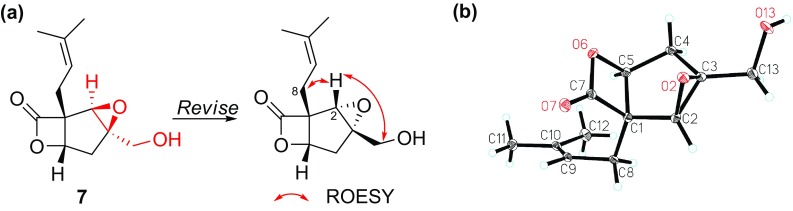
**a** Structure revision of vibralactone B (**7**); **b** ORTEP drawing of compound **7**

The previously described compound vibralactone B (**7**) possessing an epoxy ring at C2–C3 was re-encountered in this research with a yield of more than 3 g [6]. Re-examined of the ROESY spectrum suggested that the mis-assignment of configuration of the epoxy ring as supported by the obvious ROESY correlations between H-8/H-2, and H-2/H-13 (Fig. 5), suggestive of *α* configuration instead of *β* configuration of the epoxy ring. Finally, crystals of **7** suitable for single crystal X-ray diffraction analysis were cultured via slowly evaporated methanol. As shown in Fig. 5, the X-ray diffraction result unequivocally determined the epoxy ring as *α* configuration.

In conclusion, six previously undescribed compounds were isolated from the scale-up fermentation of the fungus *B. vibrans*. All these compounds are biosynthetically related. Vibralactamide A (**1**) represents the other type of nitrogen-containing vibralactone derivative possible originated from different nitrogen atom-introducing pathway comparing with those of vibralactoximes A–P. This work provided further evidences for divergent vibralactone biosynthesis pathway and expanded the structural diversity of vibralactone-related compounds.

## Experimental Section

### General Experimental Procedures

Optical rotations were obtained on a JASCO P-1020 digital polarimeter (Horiba, Kyoto, Japan). UV spectra were recorded on a Shimadzu UV-2401PC UV–visible recording spectrophotometer (Shimadzu, Kyoto, Japan). 1D and 2D NMR spectra were obtained on a Bruker Avance III 600 MHz spectrometer (Bruker Corporation, Karlsruhe, Germany). HRESIMS were recorded on an Agilent 6200 Q-TOF MS system (Agilent Technologies, Santa Clara, CA, USA). HREIMS were obtained on a Waters Autospec Premier P776 mass spectrometer. Single crystal X-ray diffraction was performed on an APEX II DUO spectrophotometer (Bruker AXS GmbH, Karlsruhe, Germany). Sephadex LH-20 (Amersham Biosciences, Uppsala, Sweden) and silica gel (Qingdao Haiyang Chemical Co., Ltd) were used for column chromatography (CC). Medium pressure liquid chromatography (MPLC) was performed on a Büchi Sepacore System equipped with pump manager C-615, pump modules C-605 and fraction collector C−660 (Büchi Labortechnik AG, Flawil, Switzerland), and columns packed with Chromatorex C-18 (40–75 μm, Fuji Silysia Chemical Ltd., Kasugai, Japan). Preparative high performance liquid chromatography (prep-HPLC) were performed on an Agilent 1260 liquid chromatography system equipped with Zorbax SB−C18 column (particle size 5 μm, dimension 9.4 mm × 150 mm, flow rate 8 mL/min, respectively) and a DAD detector (Agilent Technologies, Santa Clara, CA, USA).

### Fungal Material and Cultivation

The fungus *B. vibrans* was collected from the Ailao Mountains of Yunnan Province, P. R. China in August 2005, and identified by Prof. Mu Zang (Kunming Institute of Botany, CAS), who is a mushroom specialist. A voucher specimen of *B. vibrans* was deposited in the Herbarium of KIB. The culture medium to ferment this fungus consist of 5% glucose, 0.15% peptone from porcine meat, 0.5% yeast extract, 0.05% KH_2_PO_4_, and 0.05% MgSO_4_. Five hundred 500-mL Erlenmeyer flasks each containing 350 mL of culture medium were inoculated with *B. vibrans* strains. The incubation was carried out on rotary shakers at 24 °C and 150 rpm for 25 days in dark environment.

### Extraction and Isolation

The culture broth (175 L) of *B. vibrans* was filtered. The filtrate was concentrated under pressure to 10 L and then partition with EtOAc for four times to give a filtrate-originated EtOAc layer (170 g). Meanwhile, the mycelia were extracted by EtOH (95%) for three times. The extraction was concentrated under pressure to remove organic solvent and then dissolve in water and partition with EtOAc for four times to give a mycelium-originated EtOAc layer (183 g). The total EtOAc residue was subjected to CC over silica gel (200–300 mesh) eluting with a gradient of petroleum ether/acetone (100:0 → 0:100) to give five fractions (A–E). Fraction D was separated by MPLC eluting with MeOH/H_2_O (20:80 → 100:0) to yield ten subfractions D1–D10. Subfraction D4 was subjected to HPLC to give compound **1** (1.5 mg, MeCN/H_2_O, 25–45%, 25 min, 8 mL/min, t_*R*_ = 10.6 min). Subfraction D5 was subjected to HPLC (MeCN/H_2_O, 30–50%, 25 min, 8 mL/min) to give compounds **2** (20.5 mg, t_*R*_ = 16.5 min) and **3** (6.5 mg, t_*R*_ = 18.6 min). Subfraction D6 was separated by HPLC (MeCN/H_2_O, 33–53%, 25 min, 8 mL/min) to afford compounds **5** (30.3 mg, t_*R*_ = 15.6 min) and **6** (2.0 mg, t_*R*_ = 16.5 min). Subfraction D8 was purified by HPLC to give compound **4** (3.0 mg, MeCN/H_2_O, 36–56%, 25 min, 8 mL/min, t_*R*_ = 17.5 min).

*Vibralactamide A (* ***1*** *)* colorless oil; $\left[ \alpha \right]_{\text{D}}^{25}$ +4.2 (*c* 0.11, MeOH); UV (MeOH) λ_max_ (log *ε*) 204.5 (4.80); IR (KBr) *ν*_max_ 3447, 2975, 2972, 1637, 1383, 1126, 1044 cm^−1^, ^1^H NMR (600 MHz, acetone-*d*_6_ or DMSO-*d*_6_) and ^13^C NMR (150 MHz, acetone-*d*_6_ or DMSO-*d*_6_) data, see Tables 1 and 2; HREIMS *m/z* 253.1672 [M]^+^ (calcd for 253.1678, C_14_H_23_O_3_N).

*Vibralactone T (* ***2*** *)* colorless oil; $\left[ \alpha \right]_{\text{D}}^{25}$ −61.3 (*c* 0.08, MeOH); UV (MeOH) λ_max_ (log *ε*) 204.5 (3.53); IR (KBr) *ν*_max_ 3423, 2972, 2927, 2863, 1816, 1722, 1630, 1379, 1122, 977 cm^−1^; ^1^H NMR (600 MHz, CD_3_OD) and ^13^C NMR (150 MHz, CD_3_OD) data, see Tables 1 and 2; HRESIMS *m/z* 247.0937 [M + Na]^+^ (calcd for 247.0941, C_12_H_16_O_4_Na).

*13*-*O*-*Lactyl vibralactone (* ***3*** *)* colorless oil; $\left[ \alpha \right]_{\text{D}}^{25}$ −100.8 (*c* 0.08, MeOH); UV (MeOH) λ_max_ (log *ε*) 203.6 (3.71), 217.0 (sh, 3.50); IR (KBr) *ν*_max_ 3427, 2971, 2927, 2858, 1819, 1738, 1631, 1213, 1131, 1035 cm^−1^; ^1^H NMR (600 MHz, CD_3_OD) and ^13^C NMR (150 MHz, CD_3_OD) data, see Tables 1 and 2; HRESIMS *m/z* 303.1206 [M + Na]^+^ (calcd for 303.1203, C_15_H_20_O_5_Na).

*10*-*O*-*Acetyl vibralactone G (* ***4*** *)* colorless oil; $\left[ \alpha \right]_{\text{D}}^{25}$ −10.6 (*c* 0.18, MeOH); UV (MeOH) λ_max_ (log *ε*) 206.0 (3.01), 257.0 (sh, 2.53); IR (KBr) *ν*_max_ 3426, 2973, 2930, 2858, 1746, 1632, 1381, 1245, 1044 cm^−1^; ^1^H NMR (600 MHz, acetone-*d*_6_) and ^13^C NMR (150 MHz, acetone-*d*_6_) data, see Tables 1 and 2; HRESIMS *m/z* 249.1100 [M + Na]^+^ (calcd for 249.1097, C_12_H_18_O_4_Na).

*(11R,12R)*-*Vibradiol (* ***5*** *)* white powder; $\left[ \alpha \right]_{\text{D}}^{25}$ +9.3 (*c* 0.15, MeOH); UV (MeOH) λ_max_ (log *ε*) 196.5 (4.07), 223.0 (4.51), 264.5 (3.55), 311.5 (3.42); IR (KBr) *ν*_max_ 3449, 2977, 2925, 1637, 1384, 1262, 1126 cm^−1^; ^1^H NMR (600 MHz, acetone-*d*_6_) and ^13^C NMR (150 MHz, acetone-*d*_6_) data, see Tables 1 and 2; HREIMS *m/z* 234.1264 [M]^+^ (calcd for 234.1256, C_14_H_18_O_3_).

*(11S,12R)*-*Vibradiol (* ***6*** *)* white powder; $\left[ \alpha \right]_{\text{D}}^{25}$ +52.4 (*c* 0.15, MeOH); UV (MeOH) λ_max_ (log *ε*) 222.5 (4.45), 264.5 (3.48), 310.5 (3.35); IR (KBr) *ν*_max_ 3443, 2974, 2926, 2857, 1634, 1383, 1125, 1037 cm^−1^; ^1^H NMR (600 MHz, CD_3_OD) and ^13^C NMR (150 MHz, CD_3_OD) data, see Tables 1 and 2; HREIMS *m/z* 234.1255 [M]^+^ (calcd for 234.1256, C_14_H_18_O_3_).

*Single crystal X*-*ray diffraction data for vibralactone B (* ***7*** *, MeOH)* Crystal data for Cu_**7**_0 m: C_12_H_16_O_4_, *M* = 224.25, *a* = 10.0771(4) Å, *b* = 5.8671(2) Å, *c* = 10.3263(4) Å, *α* = 90°, *β* = 115.1150(10)°, *γ* = 90°, *V* = 552.80(4) Å^3^, *T* = 100(2) K, space group *P*21, *Z* = 2, *μ*(CuKα) = 0.834 mm^−1^, 5458 reflections measured, 1862 independent reflections (*R*_*int*_ = 0.0365). The final *R*_*1*_ values were 0.0353 (*I* > 2*σ*(*I*)). The final *wR*(*F* ^2^) values were 0.0961 (*I* > 2*σ*(*I*)). The final *R*_*1*_ values were 0.0354 (all data). The final *wR*(*F* ^2^) values were 0.0963 (all data). The goodness of fit on *F* ^2^ was 1.113. Flack parameter = 0.07(6). Crystallographic data for compound **7** have been deposited to the Cambridge Crystallographic Data Center (No. CCDC 1580061). These data can be obtained free of charge via http://www.ccdc.cam.ac.uk/conts/retrieving.html.

## Electronic supplementary material

Below is the link to the electronic supplementary material.

Supplementary material 1 (DOCX 11588 kb)
