# Supplementary material for: Vibralactone Biogenesis-Associated Analogues from Submerged Cultures of the Fungus Boreostereum vibrans
Source: Nat Prod Bioprospect. 2017 Dec 5;8(1):37–45. doi: 10.1007/s13659-017-0147-5 (PMC5803143; doi:10.1007/s13659-017-0147-5)

**Supplementary material for**

**Vibralactone Biogenesis-associated Analogues from Submerged Cultures of the Fungus *Boreostereum vibrans***

He-Ping Chen,^a^ Meng-Yuan Jiang,^b^ Zhen-Zhu Zhao,^a^ Tao Feng,^a^ Zheng-Hui Li,^a^ Ji-Kai Liu*^,a^

^a^School of Pharmaceutical Sciences, South-Central University for Nationalities, Wuhan 430074, People’s Republic of China

^b^Key Laboratory of Chemistry in Ethnic Medicinal Resources, State Ethnic Affairs Commission & Ministry of Education, School of Ethnic Medicine, Yunnan Minzu University, Kunming 650504, People’s Republic of China

Corresponding Author:

*Tel: +86-27-67842267. E-mail: [jkliu@mail.kib.ac.cn](mailto:jkliu@mail.kib.ac.cn)

**Contents**

[**Figure S1.** ^1^H NMR spectrum of **1** (600 MHz, acetone-*d*_6_). 3](#_Toc497901120)

[**Figure S2.** ^13^C and DEPT NMR spectra of **1** (150 MHz, acetone-*d*_6_). 3](#_Toc497901121)

[**Figure S3.** HSQC spectrum of **1**. 4](#_Toc497901122)

[**Figure S4.** ^1^H-^1^H COSY spectrum of **1**. 4](#_Toc497901123)

[**Figure S5.** HMBC spectrum of **1**. 5](#_Toc497901124)

[**Figure S6.** ROESY spectrum of **1**. 5](#_Toc497901125)

[**Figure S7.** ^1^H NMR spectrum of **1** (600 MHz, DMSO-*d*_6_). 6](#_Toc497901126)

[**Figure S8.** ^13^C NMR spectrum of **1** (150 MHz, DMSO-*d*_6_). 6](#_Toc497901127)

[**Figure S9.** HMBC spectrum of **1** in DMSO-*d*_6_. 7](#_Toc497901128)

[**Figure S10.** HREIMS report of **1**. 8](#_Toc497901129)

[**Figure S11.** ^1^H NMR spectrum of **2** (600 MHz, acetone-*d*_6_) 9](#_Toc497901130)

[**Figure S12.** ^13^C and DEPT NMR spectra of **2** (150 MHz, acetone-*d*_6_) 9](#_Toc497901131)

[**Figure S13.** HSQC spectrum of **2**. 10](#_Toc497901132)

[**Figure S14.** ^1^H-^1^H COSY spectrum of **2**. 10](#_Toc497901133)

[**Figure S15.** HMBC spectrum of **2**. 11](#_Toc497901134)

[**Figure S16.** ROESY spectrum of **2**. 11](#_Toc497901135)

[**Figure S17.** HRESIMS report of **2**. 12](#_Toc497901136)

[**Figure S18.** ^1^H NMR spectrum of **3** (600 MHz, CD_3_OD). 13](#_Toc497901137)

[**Figure S19.** ^13^C and DEPT NMR spectra of **3** (150 MHz, CDCl_3_). 13](#_Toc497901138)

[**Figure S20.** HSQC spectrum of **3**. 14](#_Toc497901139)

[**Figure S21.** ^1^H-^1^H COSY spectrum of **3**. 14](#_Toc497901140)

[**Figure S22.** HMBC spectrum of **3**. 15](#_Toc497901141)

[**Figure S23.** ROESY spectrum of **3**. 15](#_Toc497901142)

[**Figure S24.** HRESIMS report of **3**. 16](#_Toc497901143)

[**Figure S25.** ^1^H NMR spectrum of **4** (800 MHz, acetone-*d*_6_). 17](#_Toc497901144)

[**Figure S26.** ^13^C and DEPT NMR spectra of **4** (200 MHz, acetone-*d*_6_). 17](#_Toc497901145)

[**Figure S27.** HSQC spectrum of **4**. 18](#_Toc497901146)

[**Figure S28.** ^1^H-^1^H COSY spectrum of **4**. 18](#_Toc497901147)

[**Figure S29.** HMBC spectrum of **4**. 19](#_Toc497901148)

[**Figure S30.** ROESY spectrum of **4**. 19](#_Toc497901149)

[**Figure S31.** HRESIMS report of **4**. 20](#_Toc497901150)

[**Figure S32.** ^1^H NMR spectrum of **5** (600 MHz, CD_3_OD). 21](#_Toc497901151)

[**Figure S33.** ^13^C and DEPT NMR spectra of **5** (150 MHz, CDCl_3_). 21](#_Toc497901152)

[**Figure S34.** HSQC spectrum of **5**. 22](#_Toc497901153)

[**Figure S35.** ^1^H-^1^H COSY spectrum of **5**. 22](#_Toc497901154)

[**Figure S36.** HMBC spectrum of **5**. 23](#_Toc497901155)

[**Figure S37.** ROESY spectrum of **5**. 23](#_Toc497901156)

[**Figure S38.** HREIMS report of **5**. 24](#_Toc497901157)

[**Figure S39.** CD spectrum of compound **5**/Mo_2_(OAc)_4_ complex. 25](#_Toc497901158)

[**Figure S40.** ^1^H NMR spectrum of **6** (600 MHz, acetone-*d*_6_). 26](#_Toc497901159)

[**Figure S41.** ^13^C and DEPT NMR spectra of **6** (150 MHz, acetone-*d*_6_). 26](#_Toc497901160)

[**Figure S42.** HSQC spectrum of **6**. 27](#_Toc497901161)

[**Figure S43.** ^1^H-^1^H COSY spectrum of **6**. 27](#_Toc497901162)

[**Figure S44.** HMBC spectrum of **6**. 28](#_Toc497901163)

[**Figure S45.** ROESY spectrum of **6**. 28](#_Toc497901164)

[**Figure S46.** HREIMS report of **6**. 29](#_Toc497901165)

[**Figure S47.** CD spectrum of compound **6**/Mo_2_(OAc)_4_ complex. 30](#_Toc497901166)

[**Figure S48.** ^1^H NMR spectrum of **7** (400 MHz, CDCl_3_). 31](#_Toc497901167)

## Figure S1. ^1^H NMR spectrum of 1 (600 MHz, acetone-*d*_6_).

## Figure S2. ^13^C and DEPT NMR spectra of 1 (150 MHz, acetone-*d*_6_).

## Figure S3. HSQC spectrum of 1.

## Figure S4. ^1^H-^1^H COSY spectrum of 1.

## Figure S5. HMBC spectrum of 1.

## Figure S6. ROESY spectrum of 1.

## Figure S7. ^1^H NMR spectrum of 1 (600 MHz, DMSO-*d*_6_).

## Figure S8. ^13^C NMR spectrum of 1 (150 MHz, DMSO-*d*_6_).

## Figure S9. HMBC spectrum of 1 in DMSO-*d*_6_.

## Figure S10. HREIMS report of 1.


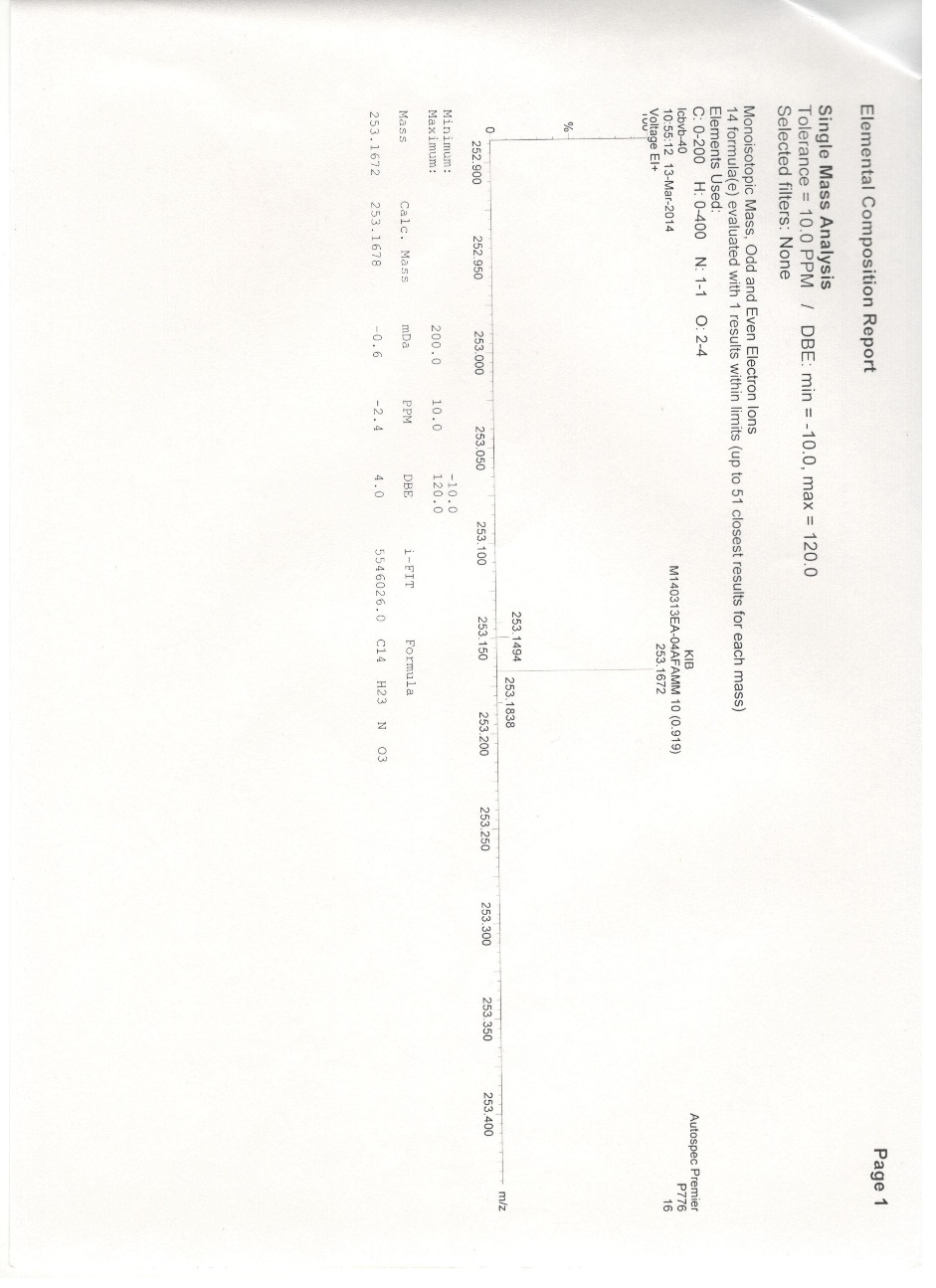


## Figure S11. ^1^H NMR spectrum of 2 (600 MHz, acetone-*d*_6_)

## Figure S12. ^13^C and DEPT NMR spectra of 2 (150 MHz, acetone-*d*_6_)

## Figure S13. HSQC spectrum of 2.

## Figure S14. ^1^H-^1^H COSY spectrum of 2.

## Figure S15. HMBC spectrum of 2.

## Figure S16. ROESY spectrum of 2.

## Figure S17. HRESIMS report of 2.


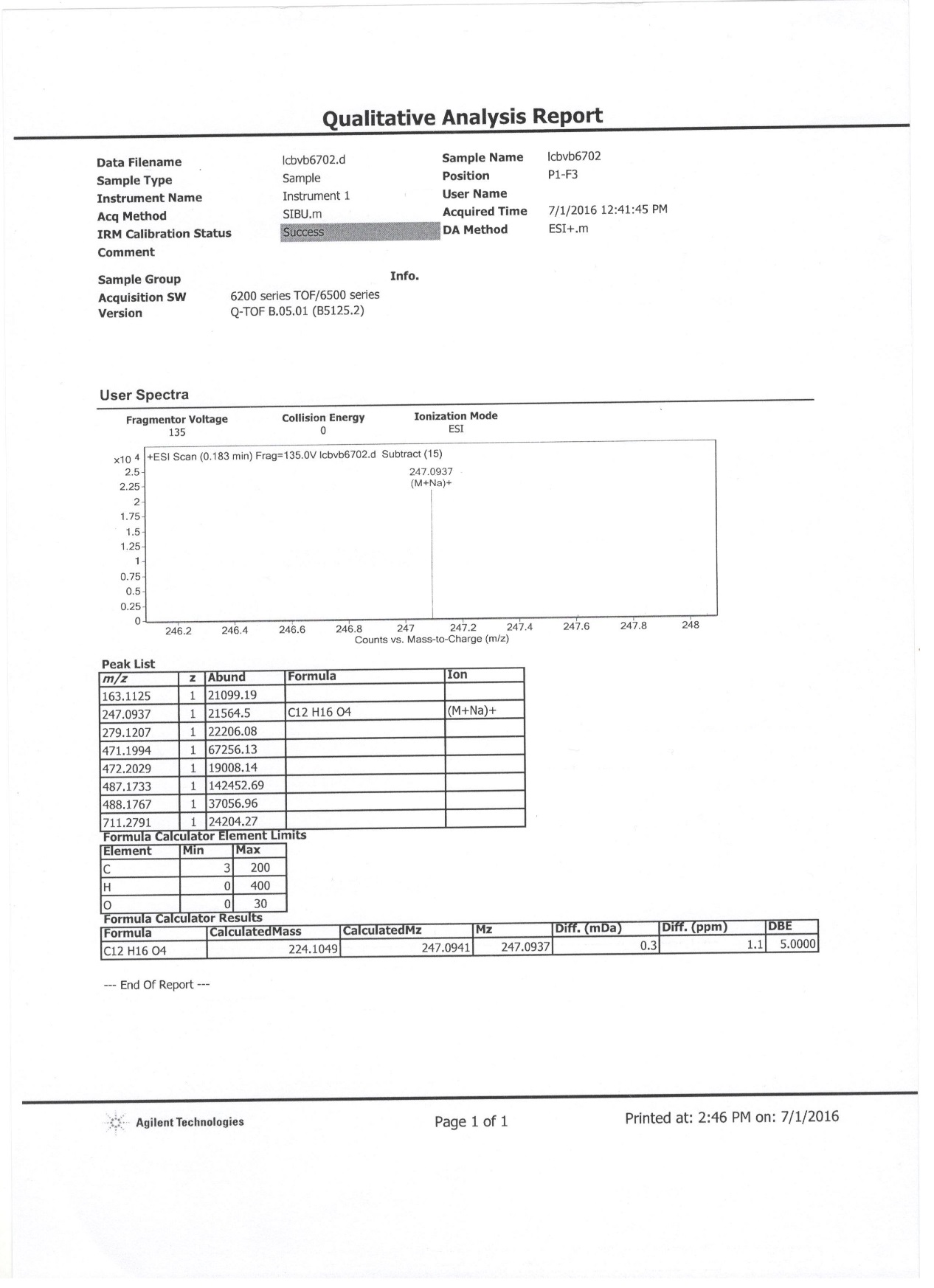


## Figure S18. ^1^H NMR spectrum of 3 (600 MHz, CD_3_OD).

## Figure S19. ^13^C and DEPT NMR spectra of 3 (150 MHz, CDCl_3_).

## Figure S20. HSQC spectrum of 3.

## Figure S21. ^1^H-^1^H COSY spectrum of 3.

## Figure S22. HMBC spectrum of 3.

## Figure S23. ROESY spectrum of 3.

## Figure S24. HRESIMS report of 3.


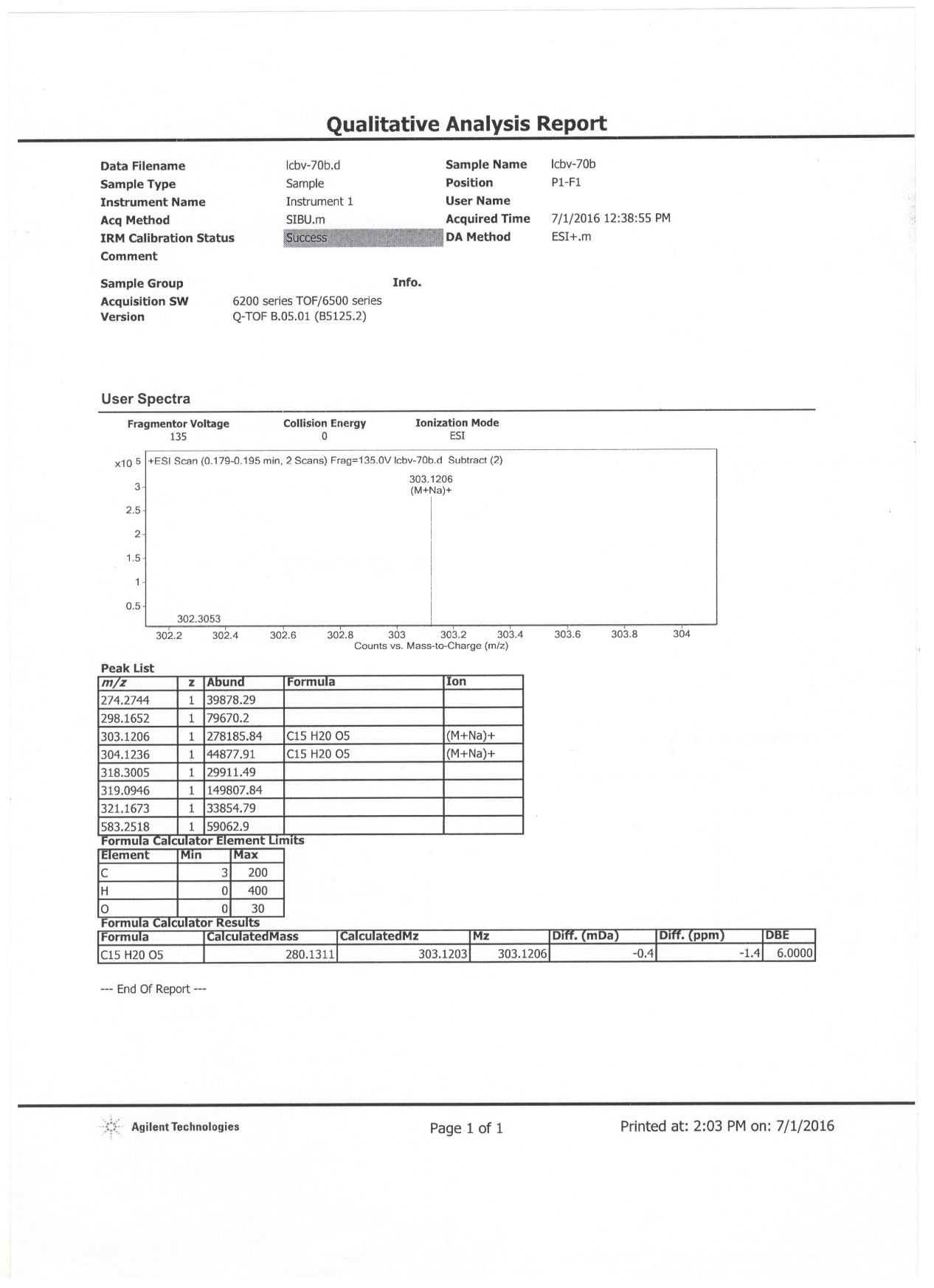


## Figure S25. ^1^H NMR spectrum of 4 (800 MHz, acetone-*d*_6_).

## Figure S26. ^13^C and DEPT NMR spectra of 4 (200 MHz, acetone-*d*_6_).

## Figure S27. HSQC spectrum of 4.

## Figure S28. ^1^H-^1^H COSY spectrum of 4.

## Figure S29. HMBC spectrum of 4.

## Figure S30. ROESY spectrum of 4.

## Figure S31. HRESIMS report of 4.


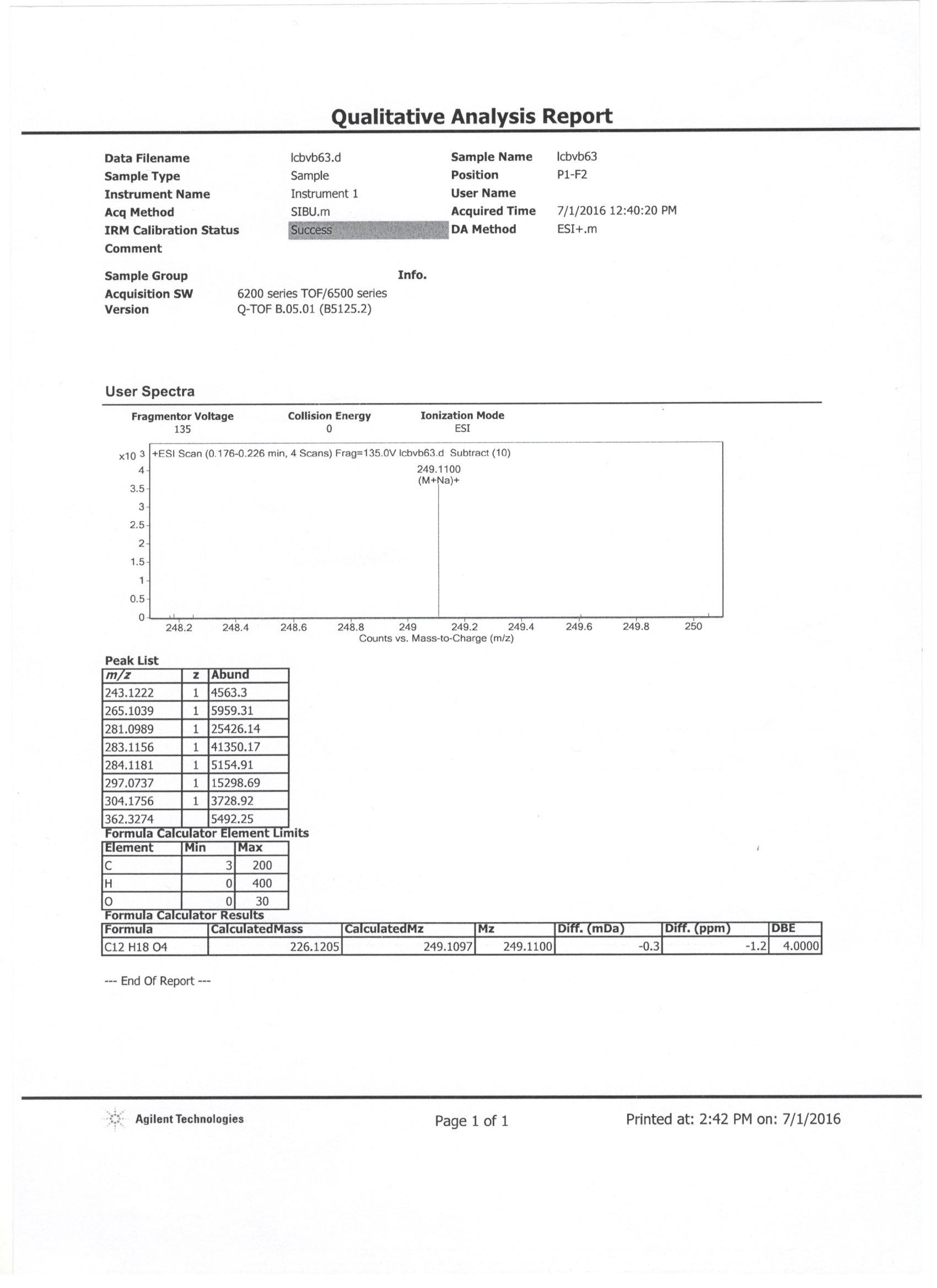


## Figure S32. ^1^H NMR spectrum of 5 (600 MHz, CD_3_OD).

## Figure S33. ^13^C and DEPT NMR spectra of 5 (150 MHz, CDCl_3_).

## Figure S34. HSQC spectrum of 5.

## Figure S35. ^1^H-^1^H COSY spectrum of 5.

## Figure S36. HMBC spectrum of 5.

## Figure S37. ROESY spectrum of 5.

## Figure S38. HREIMS report of 5.


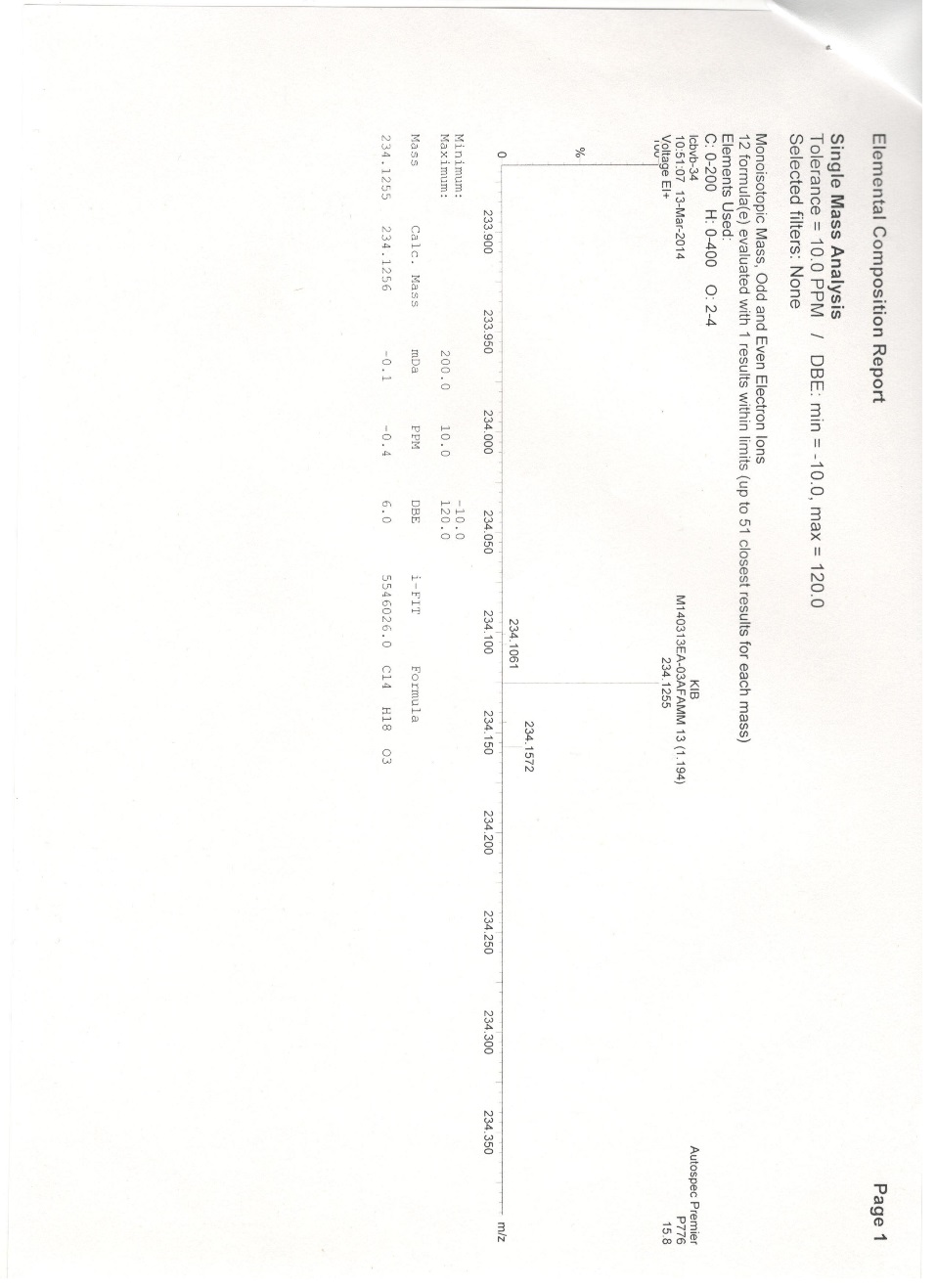


## Figure S39. CD spectrum of compound 5/Mo_2_(OAc)_4_ complex.


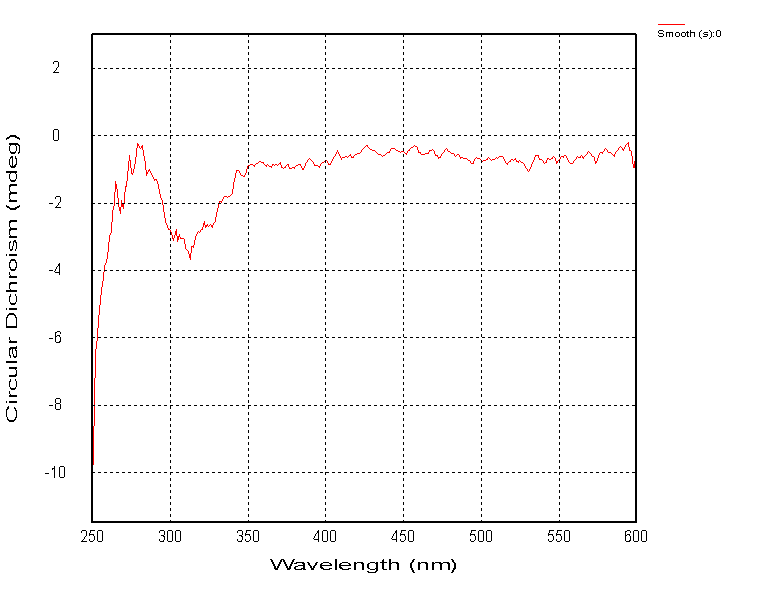


## Figure S40. ^1^H NMR spectrum of 6 (600 MHz, acetone-*d*_6_).

## Figure S41. ^13^C and DEPT NMR spectra of 6 (150 MHz, acetone-*d*_6_).

## Figure S42. HSQC spectrum of 6.

## Figure S43. ^1^H-^1^H COSY spectrum of 6.

## Figure S44. HMBC spectrum of 6.

## Figure S45. ROESY spectrum of 6.

## Figure S46. HREIMS report of 6.


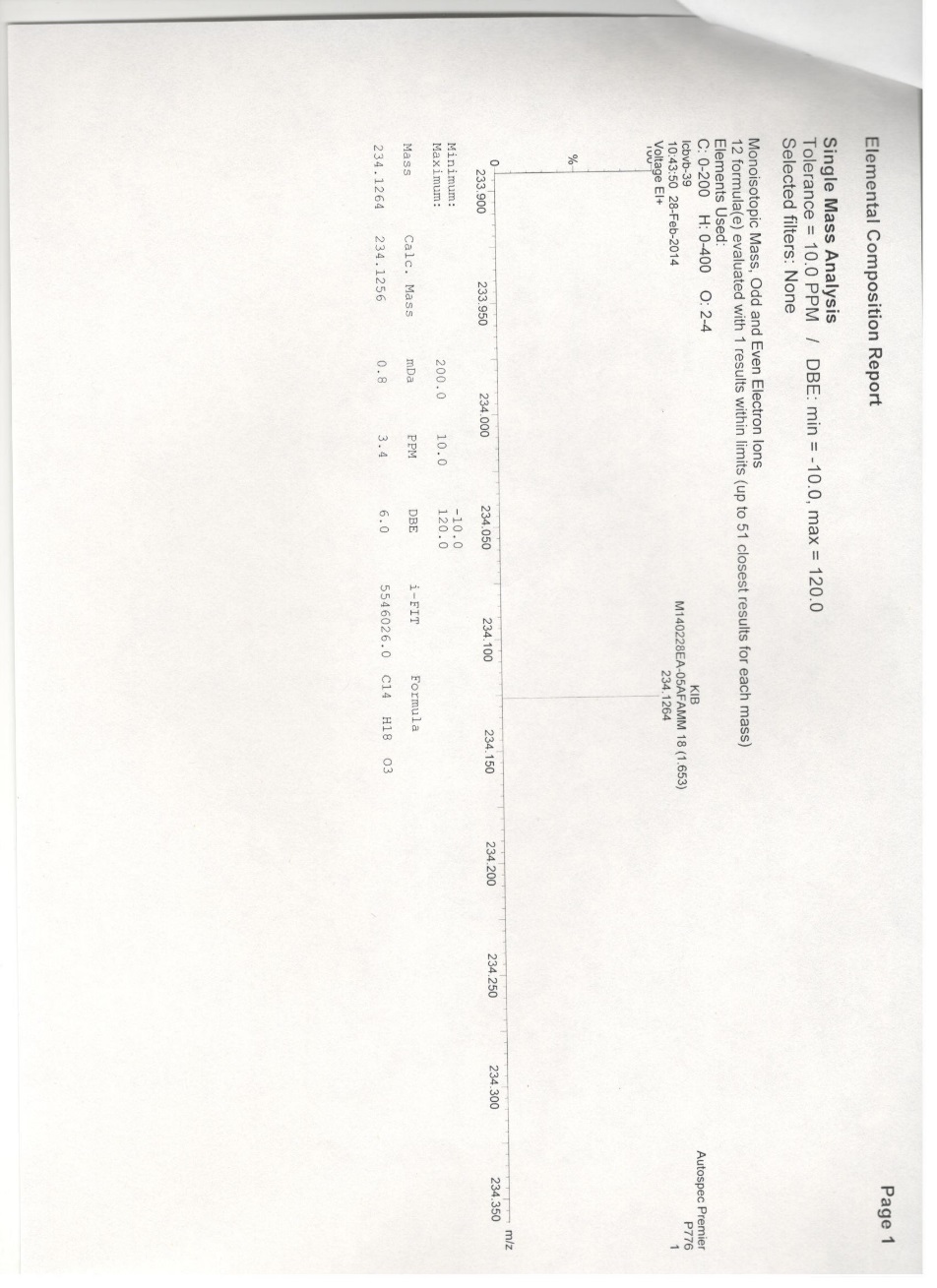


## Figure S47. CD spectrum of compound 6/Mo_2_(OAc)_4_ complex.


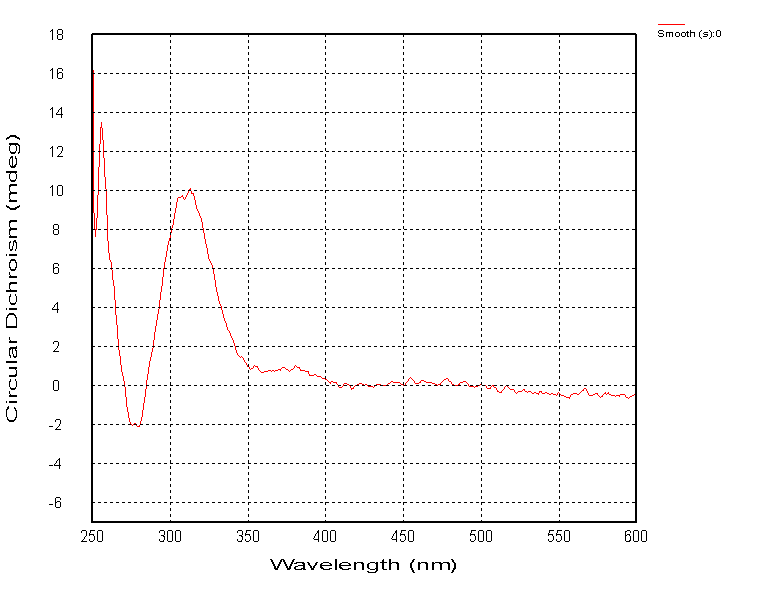


## Figure S48. ^1^H NMR spectrum of 7 (400 MHz, CDCl_3_).


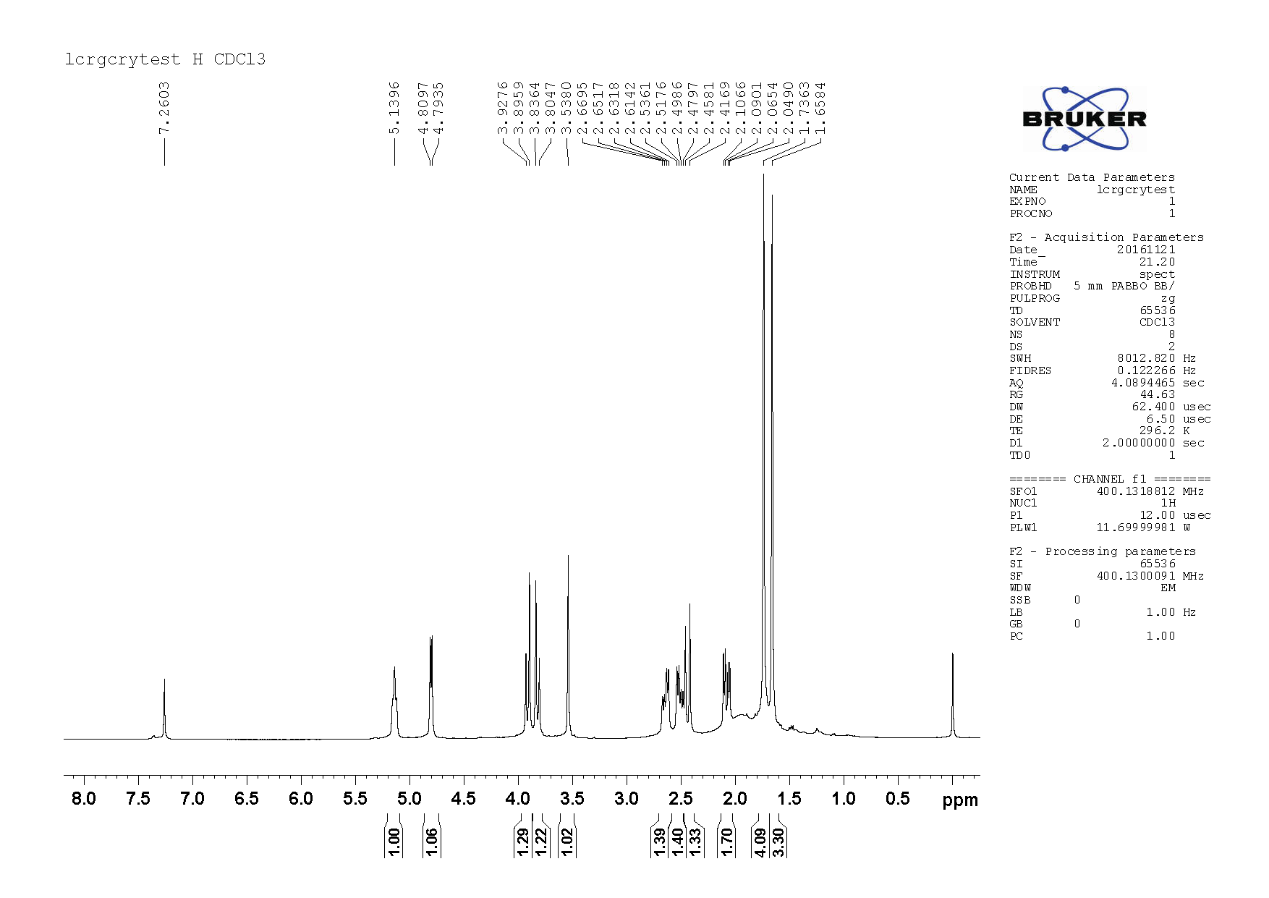

Supplement: Supplementary file 1 — Supplementary material 1 (DOCX 11588 kb) [file 13659_2017_147_MOESM1_ESM.docx]
